# Changes in Physical Activity and Sedentary Behavior before and during the COVID-19 Pandemic: A Swedish Population Study

**DOI:** 10.3390/ijerph19052558

**Published:** 2022-02-23

**Authors:** Maria Elvén, Birgitta Kerstis, Jonas Stier, Charlotta Hellström, Petra von Heideken Wågert, Micael Dahlen, Daniel Lindberg

**Affiliations:** 1 Division of Physiotherapy, School of Health, Care and Social Welfare, Mälardalen University, 72123 Vasteras, Sweden; petra.heideken.wagert@mdh.se; 2 Division of Caring Sciences, School of Health, Care and Social Welfare, Mälardalen University, 72123 Vasteras, Sweden; birgitta.kerstis@mdh.se; 3 Division of Social Work, School of Health, Care and Social Welfare, Mälardalen University, 72123 Vasteras, Sweden; jonas.stier@mdh.se (J.S.); daniel.lindberg@mdh.se (D.L.); 4 Division of Public Health Sciences, School of Health, Care and Social Welfare, Mälardalen University, 72123 Vasteras, Sweden; charlotta.hellstrom@mdh.se; 5 Department of Marketing and Strategy, Stockholm School of Economics, 11383 Stockholm, Sweden; micael.dahlen@hhs.se

**Keywords:** COVID-19, physical activity, sedentary behavior, behavior change

## Abstract

Governments have enforced measures to limit the spread of COVID-19 with varying degrees of success, which could affect people’s physical activity (PA) and sedentary behavior. This study aimed to examine changes in PA levels, types of PA, and sedentary behavior in the Swedish population before and during the COVID-19 pandemic. Associations between changed PA levels and demographical and behavioral determinants were also investigated. In December 2020, 1035 individuals (18–79 years old) completed a survey about their PA and sedentary behavior before and during the pandemic. Factors influencing their PA were also explored. Fifty-one percent of the sample reported reduced total PA, 18% had no change, and 31% increased their PA. Overall, organized PA decreased the most and sedentary behavior increased. The youngest and oldest age groups reported the greatest reduction in PA, while middle-aged groups reported the most increased PA. Men reported a larger increase in sedentary behavior than women. Mental and physical capability was associated with change in PA. In conclusion, this study indicates that, during the COVID-19 pandemic, the majority of the Swedish population have decreased PA levels with a concurrent increase in sedentary behavior, which may have negative health consequences. Interventions are recommended to address both PA and sedentary behavior, specifically to strengthen people’s ability to perform PA and focusing on the youngest and oldest age groups.

## 1. Introduction

Despite the proven health benefits of physical activity (PA), the world has struggled with a physical inactivity pandemic for decades [1]. In 2016, more than a quarter of the world’s adult population was insufficiently physically active [2]. Physical inactivity is one of the leading risk factors for non-communicable diseases (e.g., cardiovascular disease, diabetes, and chronic respiratory diseases) and death worldwide [3]. In addition, sedentary behavior (i.e., sitting, reclining, or lying postures, except while sleeping) [4] is increasing [5]. Previous research suggests that sedentary behavior is distinct from physical inactivity [6,7] and should be considered as an independent risk factor for non-communicable diseases and all-cause mortality [8]. To tackle this problem, the World Health Organization (WHO) 2020 guidelines provide recommendations on PA and sedentary behavior [9]. The WHO has also set a goal to reduce the prevalence of physical inactivity by 15% in the year 2030 [1]. However, the global PA goal will not be met if the current trends continue [2].

In March 2020, the WHO declared the spread of the novel coronavirus disease 2019 (COVID-19) to be a global pandemic, which has resulted in significant negative health consequences with 5.5 million deaths worldwide (as of January 2022) [10]. In light of the pandemic, recent studies have demonstrated that PA plays an important role in reducing the impact of the COVID-19 pandemic because it increases resistance and reduces the risk of infectious diseases and mortality, in addition to enhancing vaccination outcomes [11]. PA during the pandemic is also associated with psychological well-being [12].

Governments worldwide have enforced a range of measures to restrain the spread of COVID-19 to varying degrees, including recommendations, mandates, quarantines, and partial or total lockdowns [13]. Compared to many other countries, Sweden has implemented fewer regulations and far-reaching interventions. Instead, Sweden largely relies on recommendations, voluntary measures, and individual responsibility [14]. For example, Swedes have been recommended to work from home, distance teaching has been implemented in elementary schools and universities, and restrictions for physical/social distancing and social gatherings have been applied. These social distancing restrictions have impacted sports training and exercise at fitness centers [15].

When the COVID-19 pandemic began, people’s daily number of steps decreased significantly worldwide [16]. In April 2020, an international survey using self-report measurements based on recall demonstrated an overall PA decrease of 38%, including walking and vigorous- and moderate-intensity activities. In addition, the daily time sitting increased from 5 to 8 h. The survey was predominantly conducted in European, North African, and West Asian countries [17]. A systematic review including 22 international, retrospective and prospective studies based on self-reported data conducted until March 2021 demonstrated that older adults (age > 50 years) had significantly decreased their PA during the pandemic [18]. People in the United States who worked from home reported more time sitting than those who continued to work on-site based on self-report measurements and recall [19]. A systematic review and meta-analysis including 64 studies, using both subjective and objective measurements, conducted during year 2020 in European (Sweden not included), North and South American, Asian, and African countries revealed an increase in sedentary time during the pandemic irrespective of sex, age, and lockdown [20]. A Canadian study including self-report measurements of current PA found that people who were already physically inactive had the greatest decrease in their PA levels. They also reported fewer benefits and opportunities to be active, perceived more challenges to engage in PA, and were less motivated compared to more physically active people [21]. A Swedish study based on self-report measurements and recall [22], including 1318 participants aged > 18 years, of whom 89% were women, found that 36% of the participants reported increased PA, while 29% decreased PA during early autumn 2020. A larger share of those reporting a decrease in PA considered the change to be due to the pandemic. The odds for a decrease in PA were significantly higher in the oldest age group compared to the youngest [22]. Another Swedish study based on self-report measurements and recall, including 5599 working adults, showed small negative and positive changes in PA and sedentary behavior, respectively, over time during the COVID-19 pandemic. When changes in PA and sedentary behavior were present, they were more pronounced during the first wave (spring 2020) compared to the second (late autumn 2020) [23].

Moreover, the change to working from home, including reduced work-related commuter time and increased everyday life flexibility, has generated new possibilities for PA [24]. Different types of PA, such as exercising with others or alone [25] and outdoor or indoor activities [21], have been affected differently during the pandemic. Home-based exercise recommendations have commonly been used to counteract physical inactivity [26].

Understanding people’s behavior is complex. However, it becomes even more complex during new and unforeseen situations, such as the COVID-19 pandemic. To provide insights into change in PA during the pandemic, we need to understand the internal and external mechanisms that drive people’s behaviors. Michie, van Stralen and West’s [27] Capability, Opportunity, Motivation, and Behavior (COM-B) model shows that behaviors (e.g., PA) and related behavioral changes are determined by people’s capabilities (e.g., their mental and physical capacity to engage in a behavior), opportunities (e.g., external factors that influence behavior), and motivations (e.g., brain processes that energize behavior). These three constructs are key targets in behavior change interventions because they underpin behavior. In the UK, the adoption of the COM-B model provided insights into factors influencing people’s hygienic practices during the pandemic and informed behavior change interventions [28]. Therefore, the COM-B model is a suitable framework to explore the factors that are most likely to influence PA changes during the pandemic.

Overall, studies worldwide demonstrate decreases in PA and increases in sedentary behavior during the COVID-19 pandemic across various populations [18,20,29]. Notably, studies also indicate no change or even increased PA among population groups [30], which points to the need to further improve our understanding regarding variations in change in PA and sedentary behavior during the pandemic. More in-depth knowledge about the characteristics of people who have increased, decreased, or not changed their PA and sedentary behavior as well as factors that influenced their behavior changes is lacking. Therefore, the Swedish context constitutes an interesting case to better understand the determinants of PA during the pandemic. In addition, the Swedish measures to limit the spread of COVID-19 differ from other countries [31]. This fact may shed light upon some unique contextual factors impacting on PA. A few studies on PA and sedentary behavior during the pandemic have been conducted in Sweden [22,23]; however, these studies are limited to specific population groups and have not investigated factors influencing a change in PA. Therefore, we need to examine change in PA and sedentary behavior in more detail, with a more representative sample of the Swedish population, and include determinants of behavior change. Concurrently focusing on individual factors and the Swedish case could impact efforts to decrease negative health consequences due to the pandemic and address factors driving PA engagement during and after the pandemic. This study aimed to examine the changes in PA levels, types of PA, and sedentary behavior in the Swedish population before and during the COVID-19 pandemic. In addition, associations between changed PA levels and demographical and behavioral determinants were investigated.

## 2. Material and Methods

### 2.1. Recruitment of Participants and Data Collection

This cross-sectional population-based study used self-reported questionnaire data collected from participants who completed an online survey on 7 December 2020. The participants were recruited through a survey management service including 40,000 potential participants aged 18–79 years. A stratified sample of 2000 persons based on sex, age, and Swedish regions were sent invitations to participate in the study. In total, 1035 (51.7%) participants anonymously filled out the online survey. A follow-up email was sent on 11 December 2020. The study was conducted in accordance with the Declaration of Helsinki [32] and Swedish law [33]. All participants provided their informed consent to take part in the survey. All personal data were anonymized directly after the material was collected and were not accessible to the researchers in the present study.

At the time of data collection, Sweden experienced the second wave of the COVID-19 pandemic, with 337,800 confirmed cases of COVID-19 and 7745 deaths. The number of confirmed cases increased rapidly in December, reaching its peak at the turn of the year. The responsibility to reduce the spread of infection was first and foremost the responsibility of individuals. The Swedish government advocated to keep distance, stay home at the slightest symptom of COVID-19, work from home if possible, and avoid environments where congestion can occur and spend time with only a few family members or friends. The number of people in shops and fitness centers was adapted to the size of the premises. University teaching was performed by digital teaching and distance learning [34].

### 2.2. Measures

The questionnaire included questions regarding demographic data, COVID-19, PA, sedentary behavior, and COM-B beliefs. PA and sedentary behavior were measured with the International Physical Activity Questionnaire-Short Form (IPAQ-SF) [35]. The IPAQ-SF records the frequency and duration of PA during the last 7 days at four intensity levels: vigorous intensity, such as aerobics and running; moderate intensity, such as leisure cycling and gardening; walking as a proxy for light intensity; and sitting as a proxy for sedentary behavior. In addition, the same items from the original IPAQ-SF were modified to ask about the participants’ PA during a normal week in December 2019, which was 1 year before the current time as well as 3 months before the WHO declared a worldwide COVID-19 pandemic.

The participants were also asked to rate their changes in non-organized and organized PA using items adapted from the Patients’ Global Impression of Change scale [36]. Two items were used: (1) To what extent has the time you spent on PA on your own, i.e., non-organized activities in the last 7 days, changed compared with a normal week 1 year ago? Examples of PA are exercising at home, using outdoor gyms, brisk walks, and running. (2) To what extent has the time you spent on PA in organized activities in the last 7 days changed compared with a normal week 1 year ago? Examples of activities are training at a fitness center or swimming hall, playing with a sports team, and rehabilitation exercises. A 7-point response scale was used, which ranged from 1 = very much less time to 7 = very much more time.

The participants’ perceived capability, opportunity, and motivation for PA (COM-B beliefs) were measured using their responses to three items adapted from Keyworth, Epton, Goldthorpe, Calam, and Armitage [37]: (1) I believe it is important for my well-being to perform PA during the COVID-19 pandemic (response scale from 0 = strongly disagree to 10 = strongly agree); (2) Due to the COVID-19 pandemic, I feel that my ability to perform PA in the last 7 days was…: (response scale from 0 = much worse than a year ago to 10 = much better than a year ago); (3) Due to the COVID-19 pandemic, I feel that my opportunities (e.g., access to a good environment for PA, equipment, and personal support) to perform PA in the last 7 days were…: (response scale from 0 = much worse than a year ago to 10 = much better than a year ago). The questionnaire is presented as a Supplementary Material File S1.

### 2.3. Statistical Analysis

#### 2.3.1. Data Management

In this study, PA was calculated as minutes per week and transformed into metabolic equivalent minutes (METs) according to the IPAQ-SF scoring protocol [38]. Minutes per week were computed as minutes per PA session × frequency of sessions per week and transformed into METs by multiplying with the respective values for light intensity (walking) (3.3) and moderate intensity (4.0) and vigorous intensity (8.0) activities. The total METs/week were computed as the sum of light, moderate, and vigorous METs/week for a normal week 1 year ago and in the past 7 days, respectively. The difference between the total METs for a normal week 1 year ago and in the past 7 days was computed and categorized as a “decrease”, “no change”, or “increase” in PA. The age groups were divided into young adults (18–26 years old, *n* = 171, 16.5%), adults (27–49 years old, *n* = 317, 30.6%), middle-aged adults (50–65 years old, *n* = 310, 30.0%), and older adults (66–79 years old, *n* = 237, 22.9%).

#### 2.3.2. Analyses

The descriptive statistics are presented as frequencies and percentages for categorical variables and means and standard deviations for continuous variables. Spearman’s rho correlation was used to analyze the correlation between PA before and during the COVID-19 pandemic. The PA levels (METs) are presented as medians because of the skewed distribution of the data. Changes in PA are presented as the proportion (%) of participants reporting an increase, decrease, or no change in total PA and organized and non-organized PA. To analyze changes in PA and organized and non-organized PA concerning sex, we used Pearson’s chi-square and (*χ*^2^) Mann–Whitney *U* tests. For age, we used the Kruskal–Wallis and Dunn–Bonferroni post hoc tests. The Mann–Whitney *U* test was used to analyze differences in COM-B beliefs concerning sex, while Spearman’s rho was used to analyze their association with age. A hierarchical multiple regression analysis investigated associations among behavioral and demographical determinants and changes in total PA. The dependent variable was changes in PA, which were categorized as increased, no change, and decreased PA. The independent variables were capability, opportunity, motivation, and age. Capability was entered first in the model because this variable had the strongest bivariate correlation with changes in total PA among the behavioral determinants. Sex was excluded because there was no correlation between sex and changes in total PA. Bivariate correlation coefficients, tolerance values, and variance inflation factors were calculated to detect the possible multicollinearity among the independent variables [39]. The analyses showed no indications of multicollinearity. All tests were two-tailed and the significance level was set to *p* < 0.05. The analyses were performed using IBM SPSS Statistics (version 26.0; IBM SPSS, Armonk, NY, USA).

## 3. Results

### 3.1. Description of the Sample

The mean age of the sample was 50.6 years (SD = 16.5), 49.5% were women, and 5.3% were confirmed to have had COVID-19. The descriptive characteristics for the sample are presented in Table 1.

### 3.2. Changes in Total, Vigorous, Moderate, and Light PA

**Figure 1:**
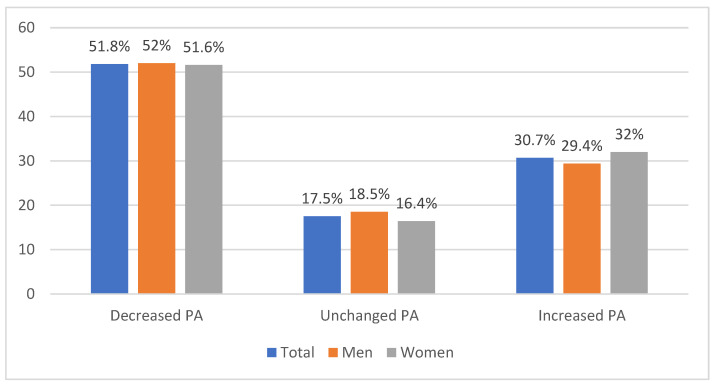
Proportion of participants reporting decreased, increased, or unchanged total PA. Note: No significant difference existed between men and women, *χ*^2^(2) = 1.251 (*p* > 0.05).

**Figure 2:**
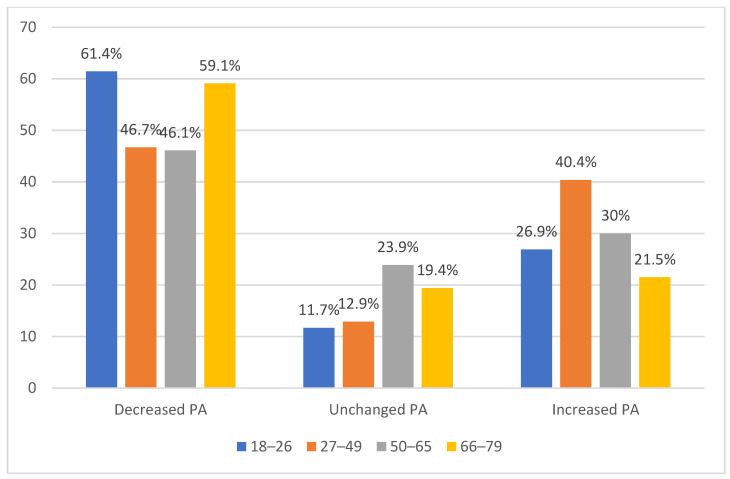
Proportion, dichotomized into age groups, of participants reporting decreased, increased, or unchanged total PA. Note: A significant difference existed between the age groups, *χ*^2^(6) = 70.772 (*p* < 0.001).

An association was found between total METs/week before and during the COVID-19 pandemic (*r*_s_ = 0.692; *p* < 0.001), vigorous METs/week before and during the pandemic (*r*_s_ = 0.653; *p* < 0.001), moderate METs/week before and during the pandemic (*r*_s_ = 0.768; *p* < 0.001), and light METs/week before and during the pandemic (*r*_s_ = 0.698; *p* < 0.001). Hence, the total PA before the pandemic was strongly associated with the total PA during the pandemic as well as vigorous, moderate and light PA before and during the pandemic. Fifty-two percent of the participants reported a decrease in total METs/week during the pandemic, while 18% reported no change and 31% reported an increase; see further details in Figure 1 and Figure 2.

In total, the decrease, increase, and no change in PA led to a decrease in total METs/week for the total sample. The vigorous and moderate METs/week also decreased compared with before the pandemic in the total sample. There was no change in light METs/week (see Table 2).

Both men and women reported decreased total, vigorous, and moderate METs/week during the pandemic compared with before the pandemic. Light METs/week did not change for either men or women. The changes in total, vigorous, moderate, and light METs/week did not differ between men and women.

All age groups reported decreased total, vigorous, and moderate METs/week during the pandemic compared with before the pandemic. Light METs/week did not change for the 18–26, 50–65, and 66–79-year-old age groups, while the 27–49-year-old age group increased their light METs/week. The change in total METs/week differed between the age groups, where the 18–26-year-old age group decreased their total PA more than the 27–49-year-old age group, while the 66–79-year-old age group decreased their total PA more than the 27–49 and 50–65-year-old age groups. All age groups decreased their vigorous METs/week, but no age group showed greater changes compared with the other age groups. The 66–79-year-old age group decreased their moderate METs/week more than the 27–49 and 50–65-year-old age groups. The 27–49-year-old age group increased their light METs/week more than the 66–79-year-old age group (see Table 2).

### 3.3. Changes in Types of PA

In relation to organized PA, 44% of participants reported a decrease during the pandemic, 29% reported the same amount, while 8% reported an increase. Twenty percent of the sample never engaged in organized PA either before or during the pandemic. Concerning non-organized PA, 35% reported a decrease during the pandemic, 34% reported the same amount, and 25% reported an increase. Six percent of the sample never engaged in non-organized PA either before or during the pandemic. There was an association between the changes in total METs/week and changes in non-organized PA (*r*_s_ = 0.328; *p <* 0.01) and in changes in organized PA (*r*_s_ = 0.213; *p* < 0.01). Hence, a decrease in total PA was associated with a decrease in organized and non-organized PA, and vice versa. Furthermore, organized PA decreased the most compared with before the pandemic, but one-quarter of the sample simultaneously increased their non-organized PA.

Women reported a larger decrease in their organized PA than men did during the pandemic (*χ*^2^ = 18.37; *p* = 0.01). Women simultaneously reported a larger increase in non-organized PA than men did during the pandemic (*χ*^2^ = 16.83; *p* = 0.02). There were no differences in changes in organized or non-organized PA between the age groups.

### 3.4. Changes in Sedentary Behavior

Sedentary behavior increased by 1.5 h per day during the COVID-19 pandemic for the total sample. Both men and women reported an increase in sedentary behavior, but the increase among men was larger compared with the increase among women. All age groups reported an increase in sedentary behavior during the pandemic, although the change did not differ between the age groups (see Table 3).

### 3.5. Determinants of Changes in PA

People’s motivation for PA during the COVID-19 pandemic was high (*Mdn* = 9). Motivation did not show any association with sex. Motivation and age showed a weak negative association (*r*_s_ = −0.10; *p* < 0.01).

The median value for the self-perceived capability to perform PA during the pandemic was 5 out of 10. Thirty-five percent of participants rated their capability to perform PA during the pandemic as worse (ratings ≤ 4) compared with before the pandemic. Men reported better capability to perform PA (*Mdn* = 5) compared to women (*Mdn* = 5) (*U* = 120,725.5; *p* = 0.03). Capability and age showed a weakly positive association (*r_s_* = 0.08; *p* < 0.01). Altogether, the capability of the sample to perform PA was negatively affected during the pandemic, and sex and age were relevant in the participants’ capability to perform PA.

The median value for opportunities to perform PA during the pandemic was 4 out of 10. Fifty percent rated their PA opportunities during the pandemic as worse (ratings ≤ 4) compared with before the pandemic. Men reported better PA opportunities (*Mdn* = 5) compared to women (*Mdn* = 4; *U* = 109602.5; *p* < 0.001). Opportunities and age showed a weakly positive association (*r*_s_ = 0.13; *p* < 0.01). Altogether, the sample perceived worse PA opportunities during the pandemic, and sex and age were relevant in PA opportunities.

Table 4 shows that the entire regression model explained 10% of the total variance of change in total PA (METs/week). Capability alone explained 9% of the variance, while age added 1% of the variance. Opportunities and motivation did not add any proportion of the explained variance. Higher capability was associated with increased PA and higher age was associated with decreased PA, and vice versa.

## 4. Discussion

This study examined changes in PA levels, types of PA, and sedentary behavior in the Swedish population before and during the COVID-19 pandemic. In addition, associations between changed PA levels and demographical and behavioral determinants were investigated. The main findings demonstrate that every second person reduced their total PA level during the pandemic. Sedentary behavior increased by 1.5 h per day, whereas men increased their sedentary behavior the most. Both organized and non-organized PA decreased, while organized PA decreased the most, specifically among women. Considering PA levels, there were no differences based on sex, but differences were found among the age groups. The largest reduction in PA level was observed among the youngest and oldest age groups. Adults aged between 27 and 49 years increased their PA the most. The perceived capability to perform PA was most strongly associated with changes in PA. However, the results show an appreciable unexplained variance in change in PA, which suggests that other variables, as yet unidentified, also contributed to the change.

The median PA levels were relatively low compared with the WHO recommendations for PA [9] and the results of a previous Swedish study during the pandemic [22]. A potential reason for this finding could be that the data collection was carried out in December 2020, when the second wave peaked in Sweden, while the data collection in the study by Eek et al. [22] took place earlier in 2020. In addition, the different demographics of the study samples, such as a larger share of older participants in the present sample could explain some of the differences. The widely distributed PA levels in the current study sample should also be considered when comparing and interpreting the results.

To improve our understanding of why people’s behavior changes during the pandemic, we must pay attention to the range of mechanisms that can be involved in behavioral changes, including those that are related to the individual and those that involve environmental changes [40]. Better self-perceived capabilities to perform PA appeared to be associated with increased PA, and vice versa. The perceived importance of PA did not explain the changes in PA levels, which is a surprising finding because motivation is an important determinant of PA during non-pandemic circumstances [41].

Our results both support and do not support previous results explaining PA behavior during the pandemic. Among adults in the UK, opportunities and motivation were associated with shifts in PA based on self-reported measurements of current PA, while capability played a limited role in PA. Only conscious-based motivation predicted the UK adults’ PA, whereas automatic motivation based on habits did not predict their PA [42]. In the current study, participants with high or low PA levels before the pandemic continued with this pattern during the pandemic. This indicates that the Swedes’ PA habits dominated their PA during the pandemic. A likely explanation for the differences in the results might be that the UK has implemented periods of lockdown periods during the pandemic [38], which may have limited PA opportunities more in the UK than in Sweden. The association between capability and change in PA highlights how people’s mental as well as physical capacity play an important role in their PA levels during the pandemic. Previous studies have demonstrated that mental capacity could be reduced by, for example, fear of being infected, distress [43], and depression during the pandemic [44], and physical capacity by weakened aerobic capacity due to the effects of COVID-19 infection [45]. A Belgian study [25] demonstrated that fears of COVID-19 contagion was the main barrier for engaging in PA among low-activity people. Belgium, like Sweden, opted for a light lockdown where citizens were allowed to exercise in their homes or outdoors [13]. Thus, the findings of the Belgian study support the relationship between capability and PA during the pandemic. In Sweden, people’s mental and physical capacity seem to supersede motivation and PA opportunities during the pandemic. A possible reason is that Sweden has not implemented periods of total lockdown, which has given people some opportunities to engage in PA, e.g., outdoors, or together in small groups. Thus, the Swedish case shed light on the impact of people’s self-perceived capacity to perform PA, which needs to be specifically considered in interventions during and after the pandemic. A possible method for exploring this finding is to target people’s self-efficacy (belief in one’s capabilities to engage in a specific behavior) [46] to increase PA behavior as there is convincing evidence of the relationship between self-efficacy and PA [47]. Suggested strategies include providing specific recommendations for when, where, and how PA can be performed instead of generally encouraging PA. This guidance may include individually tailored support for PA in a safe environment, providing opportunities to see peers performing PA, such as virtually via social media or in real life, and providing positive feedback [48,49].

When analyzing the PA changes during the pandemic in more detail, a more nuanced pattern evolves according to participants’ age and types of PA. The current findings showing that the oldest age group decreased their overall PA level more compared with younger age groups are supported by previous studies using self-reported measurements [22,50]. This finding is concerning because PA in older age decreases the risk of several lifestyle-related diseases and comorbidity [51], while the number of sedentary hours is found to be associated with an increased risk of mortality [52]. The oldest age group had the lowest number of sedentary hours, which is surprising because sedentary time usually increases during retirement and old age [53] and is described as a natural aspect of aging [54]. Notably, the youngest age group in this study reported a high level of sedentary time during the pandemic and they experienced a greater decrease in their total PA levels compared with the adult group. These findings are similar to a Spanish study based on self-reported measurements and recall, including adults of 18–64 years, showing that the youngest age group decreased their PA and increased their sedentary time the most during the pandemic [55]. However, Spain has implemented strict rules stipulating confinement, which need to be considered when comparing results from Sweden and Spain. A US study that used direct observations of children’s PA revealed a significant decline in the number of physically active children (aged 1–12 years) at parks during the pandemic compared to before the pandemic [56]. It is important that these habits not persist over time because infrequent participation in PA at a young age is associated with physical inactivity in adulthood [57]. Furthermore, the volume of sedentary time in the young age group needs specific attention as evidence points to specific health risks of sedentary behavior even if an individual performs PA [6]. Moreover, studies have demonstrated that psychological distress increased more in younger adults than older adults during the pandemic [58]. This may be because the younger population had to decrease their social activities a lot during the pandemic to reduce the spread of infection, even though they do not have the greatest risk of serious illness and early death [59]. In addition, the already very low PA levels among younger people during non-pandemic circumstances [1] make them even more vulnerable during the pandemic because of the additional barriers. Taken together, these results emphasize the need to pay specific attention to young adults in the employment of health-improving strategies.

The consequences of COVID-19 restrictions and recommendations have not only affected PA levels, but also the patterns of PA practices. Both non-organized and organized PA decreased, but organized PA decreased the most. Constandt et al. [25] demonstrated that the loss of competitive elements when performing PA was one of the main reasons for the reduction in PA during the COVID-19 pandemic in Belgium. Competitive elements are a usual aspect of sports activities performed in teams or at fitness gyms; therefore, this could be a possible explanation for our findings. In addition, women changed their type of PA more than men did, with a larger decrease in organized PA and a larger increase in non-organized PA. Hence, the COVID-19 restrictions, such as closed or reduced activities at fitness centers, appear to have impacted women more than men. However, women appear to have adapted to these changed circumstances and developed behavior to compensate for their loss of access to fitness centers by performing more non-organized activities, such as home-based exercises. This flexibility in types of PA performance may create opportunities to incorporate PA into daily living during the current pandemic and other social health-care crises, which is essential to improving well-being [12] and preventing lifestyle-related diseases [60].

### Strengths and Limitations

The strengths of this study are its population study design and the relatively large sample, which included participants aged 18 to 79 years, which has resulted in new knowledge about the consequences for the Swedish adult population, their PA and sedentary behavior during the pandemic. Another strength is that the participants rated their PA levels twice for the past 7 days and during the corresponding period 1 year before. By measuring PA levels during the same month of the year during the pandemic and just before the pandemic broke out, the effect of weather and time on PA levels decreased. Although the relatively large population implies good statistical power, some limitations should be acknowledged. First, the cross-sectional design precludes assumptions about the temporality or causality of the association between COVID-19 and PA. Second, there are limitations related to response bias. Although IPAQ-SF is a recommended and widely used measure, the validity of its self-reported measures for PA is not optimal [61]. Additionally, uncertain data regarding PA before the pandemic due to difficulties remembering performed PA a year ago may have influenced the results. A possible solution to address this limitation in future research is the use of objective measures of PA [62], such as direct observation [56]. Third, the 48% drop-out and the overrepresentation of people with a university degree in this study (53%) compared with the Swedish population in general (29%) [63] might affect the representativeness of the sample.

## 5. Conclusions

During the COVID-19 pandemic, most of the Swedish population decreased their PA levels and concurrently increased their sedentary behavior. Drawing upon the ample literature, this may have both a short- and long-term negative health impact. The consequences of the pandemic on PA and sedentary behavior pose a challenge for the global goal of significantly increased PA. Self-perceived mental and physical capacity and age explain some of the variations in PA change. However, other variables must be studied to further understand the determinants of PA. To adapt to new circumstances during the pandemic, including societal restrictions and the risk of COVID-19 contagion, people have changed their PA behaviors by increasing their non-organized PA, e.g., home-based training, and by decreasing their organized PA, e.g., at fitness centers, which suggests that many people can act based on changed conditions. However, the overall PA decrease indicates the need for further innovations for safe and easily accessible PA during the pandemic. Innovations also need to address sedentary behavior, specifically since increased PA cannot fully replace sedentary time. Even though adaptations have been important to maintain normal PA for many, some groups need specific attention to regain their previous PA levels. Considering the strong evidence of positive health effects of PA and reduced sedentary behavior, in addition to the emerging evidence that PA can yield positive COVID-19 outcomes, the current study findings show the need for novel and additional efforts to support increased PA. The Swedish context during the pandemic shed light on the need to improve people’s self-perceived capability for PA in interventions. Furthermore, we need to focus on the youngest and oldest age groups and implement interventions on an individual basis as well as in organizational contexts. Given this cross-sectional study was conducted during the second wave of COVID-19, follow-up studies are needed to investigate the changing trends in PA during the pandemic.

## Figures and Tables

**Table 1:** Descriptive characteristics of the sample (*N* = 1035).

Characteristics	*n* (%)	*M* (*SD*)
Age		50.6 (16.6)
Sex		
Men	523 (50.5)	
Women	512 (49.5)	
Highest education		
Compulsory school (9 years)	66 (6.4)	
Senior high school	421 (40.7)	
University	546 (52.8)	
Other	2 (0.2)	
Confirmed COVID-19		
Yes	55 (5.3)	
Chronic disease		
Yes	180 (17.4)	
Occupation		
Students/parental leave	97 (9.4)	
Manual workers	218 (21.1)	
Non-manual workers/Self-employed	407 (39.3)	
Unemployed Sick leave/early retired	65 (6.3)	
Retired	248 (24.0)	
Cohabitation status		
Married/partnership	494 (47.7)	
Living with partner	217 (21.0)	
One person household/single parent	324 (31.3)	
Origin		
Born in Sweden	930 (92.5)	

**Table 2:** PA before and during the pandemic for the total sample and in relation to sex and age.

	Total PA METs/w *Mdn*				Vigorous PA METs/w *Mdn*				Moderate PA METs/w *Mdn*				Light PA METs/w *Mdn*			
	Before	During	*p*	Post Hoc	Before	During	*p*	Post Hoc	Before	During	*p*	Post hoc	Before	During	*p*	Post Hoc
Total sample ^a^	2400	1960	<0.001		480	32	<0.001		480	240	<0.001		792	792	NS	
Sex ^a^																
Men	2556	2079	0.001		480	80	0.001		720	400	0.001		693	693	NS	
Women	2317	1825	0.001		480	0	<0.001		480	240	0.001		792	808	NS	
Difference ^b^			NS				NS				NS				NS	
Age ^a^																
18–26 year	2758	1980	<0.001		960	320	0.001		528	240	0.001		693	660	NS	
27–49 year	1920	1733	<0.001		480	48	0.001		360	240	0.001		495	660	0.001	
50–65 year	2346	2007	<0.001		320	0	0.001		650	340	0.001		792	742	NS	
66–79 year	3066	2205	<0.001		320	0	0.001		720	320	0.001		1188	1155	0.05	
Difference ^c^			0.001	A > B D > B D > C			0.03				<0.001	D > B D > C			<0.001	B > D

Abbreviations: ^a^ Wilcoxon signed-rank test, ^b^ Difference in change in PA between men and women, Mann–Whitney test, ^c^ Difference in change in PA between age groups, Kruskal–Wallis test with Dunn–Bonferroni post hoc test, METs/w = metabolic equivalent minutes per week, A = age group 18–26 year, B = age group 27–49 year, C = age group 50–65 year, D = age group 66–79 year, NS = Not significant.

**Table 3:** Sedentary behavior before and during the pandemic for the total sample and in relation to sex and age.

	Sedentary Behavior Minutes/Week *Mdn* (*IQR*)		
	Before	During	*p*
Total sample ^a^	2520 (2520)	3150 (2100)	<0.001
Sex ^a^			
Men	2534 (2520)	3360 (2310)	<0.001
Women	2520 (2520)	2940 (2100)	<0.001
Difference in change in sedentary behavior between men and women ^b^			0.05
Age ^a^			
18–26 year	3360 (2310)	4095 (2940)	0.001
27–49 year	3360 (2517)	3360 (2555)	0.001
50–65 year	2520 (2100)	2940 (2100)	0.001
66–79 year	2100 (1313)	2310 (1680)	0.001
Difference in change in PA between age groups ^c^			NS

Abbreviations: ^a^ Wilcoxon signed-rank test, ^b^ Mann–Whitney test, ^c^ Kruskal–Wallis test with Dunn–Bonferroni post hoc test, IQR = Interquartile range, NS = Not significant.

**Table 4:** Hierarchical regression analysis for change in total PA (METs/week).

Parameter	B (95% CI)	SE B	*β*	*p*
Step 1				
Capability	0.13 (0.10–0.15)	0.01	0.31	<0.001
Step 2				
Capability	0.12 (0.09–0.15)	0.02	0.29	<0.001
Opportunity	0.01(−0.02–0.04)	0.02	0.02	NS
Step 3				
Capability	0.12 (0.09–0.15)	0.02	0.30	<0.001
Opportunity	0.01 (−0.02–0.04)	0.02	0.02	NS
Motivation	−0.01 (−0.03–0.02)	0.01	−0.02	NS
Step 4				
Capability	0.12 (0.09–0.15)	0.02	0.30	<0.001
Opportunity	0.01 (−0.02–0.04)	0.02	0.03	NS
Motivation	−0.01 (−0.03–0.01)	0.01	−0.02	NS
Age	−0.01 (−0.01–0.01)	0.00	−0.07	0.02

Abbreviations: B = unstandardized regression coefficient, 95% CI = 95% confidence interval, SE B = standard error, *β =* standardized beta coefficient, NS = not significant. Note. Step 1: Adjusted R^2^ = 0.09; ∆R^2^ = 0.09 (*p* < 0.001). Step 2: Adjusted R^2^ = 0.09; ∆R^2^ = 0.00 (*p* = 0.59). Step 3: Adjusted R^2^ = 0.09; ∆R^2^ = 0.00 (*p* = 0.59). Step 4: Adjusted R^2^ = 0.10; ∆R^2^ = 0.01 (*p* = 0.02)

## Data Availability

The data presented in this study are openly available in FigShare at doi:10.6084/m9.figshare.19208538.

## Supplementary Materials

The following are available online at https://www.mdpi.com/article/10.3390/ijerph19052558/s1, File S1: Questionnaire about Physical Activity and Sedentary behavior.

## References

[1] World Health Organization Global Action Plan on Physical Activity 2018–2030: More Active People for a Healthier World. https://www.who.int/publications/i/item/9789241514187.

[2] Guthold R., Stevens G.A., Riley L.M., Bull F.C. (2018). Worldwide trends in insufficient physical activity from 2001 to 2016: A pooled analysis of 358 population-based surveys with 1·9 million participants. Lancet Glob. Health.

[3] World Health Organization WHO Global Status Report on Noncommunicable Diseases 2014. https://apps.who.int/iris/bitstream/handle/10665/148114/9789241564854_eng.pdf?sequence=1.

[4] Tremblay M.S., Aubert S., Barnes J.D., Saunders T.J., Carson V., Latimer-Cheung A.E., Chastin S.F.M., Altenburg T.M., Chinapaw M.J.M. (2017). Sedentary Behavior Research Network (SBRN)—Terminology consensus project process and outcome. Int. J. Behav. Nutr. Phys. Act..

[5] Hagströmer M., Kwak L., Oja P., Sjöström M. (2015). A 6 year longitudinal study of accelerometer-measured physical activity and sedentary time in Swedish adults. J. Sci. Med. Sport..

[6] Panahi S., Tremblay A. (2018). Sedentariness and health: Is sedentary behavior more than just physical inactivity?. Front. Public Health.

[7] Van der Ploeg H.P., Hillsdon M. (2017). Is sedentary behaviour just physical inactivity by another name?. Int. J. Behav. Nutr. Phys. Act..

[8] Patel A.V., Maliniak M.L., Rees-Punia E., Matthews C.E., Gapstur S.M. (2018). Prolonged leisure time spent sitting in relation to cause-specific mortality in a large US cohort. Am. J. Epidemiol..

[9] Bull F.C., Al-Ansari S.S., Biddle S., Borodulin K., Buman M.P., Cardon G., Carty C., Chaput J.P., Chastin S., Chou R. (2020). World Health Organization 2020 guidelines on physical activity and sedentary behaviour. Br. J. Sports Med..

[10] World Health Organization WHO Coronavirus (COVID-19) Dashboard. https://covid19.who.int.

[11] Chastin S.F.M., Abaraogu U., Bourgois J.G., Dall P.M., Darnborough J., Duncan E., Dumortier J., Pavón D.J., McParland J., Roberts N.J. (2021). Effects of regular physical activity on the immune system, vaccination and risk of community-acquired infectious disease in the general population: Systematic review and meta-analysis. Sports Med..

[12] Dahlen M., Thorbjørnsen H., Sjåstad H., von Heideken Wågert P., Hellström C., Kerstis B., Lindberg D., Stier J., Elvén M. (2021). Changes in physical activity are associated with corresponding changes in psychological well-being: A pandemic case study. Int. J. Environ. Res. Public Health.

[13] Mon B., McFall-Johnsen M. A Third of the Global Population Is on Coronavirus Lockdown—Here’s Our Constantly Updated List of Countries and Restrictions. https://www.businessinsider.nl/countries-on-lockdown-coronavirus-italy-2020-3?international=true&r=US.

[14] Irwin R.E. (2020). Misinformation and de-contextualization: International media reporting on Sweden and COVID-19. Glob. Health.

[15] Swedish Government The Swedish Way: Government Policies and Practices. https://www.government.se/.

[16] Tison G.H., Avram R., Kuhar P., Abreau S., Marcus G.M., Pletcher M.J., Olgin J.E. (2020). Worldwide effect of COVID-19 on physical activity: A descriptive study. Ann. Intern. Med..

[17] Ammar A., Brach M., Trabelsi K., Chtourou H., Boukhris O., Masmoudi L., Bouaziz B., Bentlage E., How D., Ahmed M. (2020). Effects of COVID-19 home confinement on eating behaviour and physical activity: Results of the ECLB-COVID19 international online survey. Nutrients.

[18] Elisabeth A.L., Karlen S.B.-L., Magkos F. (2021). The effect of COVID-19-related lockdowns on diet and physical activity in older adults: A systematic review. Aging Dis..

[19] Lindsey B.W., Boolani A., Merrigan J.J., Cortes N., Caswell S.V., Martin J.R. (2021). Relationship between employment status, reported physical activity, and sitting time during COVID-19 pandemic. J. Phys. Act. Health.

[20] Runacres A., Mackintosh K.A., Knight R.L., Sheeran L., Thatcher R., Shelley J., McNarry M.A. (2021). Impact of the COVID-19 pandemic on sedentary time and behaviour in children and adults: A systematic review and meta-analysis. Int. J. Environ. Res. Public Health.

[21] Lesser I.A., Nienhuis C.P. (2020). The impact of COVID-19 on physical activity behavior and well-being of Canadians. Int. J. Environ. Res. Public Health.

[22] Eek F., Larsson C., Wisén A., Ekvall Hansson E. (2021). Self-perceived changes in physical activity and the relation to life satisfaction and rated physical capacity in Swedish adults during the COVID-19 pandemic—a cross sectional study. Int. J. Environ. Res. Public Health.

[23] Blom V., Lönn A., Ekblom B., Kallings. L.V., Väisanen D., Hemmingsson E., Andersson G., Wallin P., Stenling A., Ekblom Ö. (2021). Lifestyle habits and mental health in light of the two COVID-19 pandemic waves in Sweden, 2020. Int. J. Environ. Res. Public Health.

[24] Vyas L., Butakhieo N. (2020). The impact of working from home during COVID-19 on work and life domains: An exploratory study on Hong Kong. Policy Des. Pract..

[25] Constandt B., Thibaut E., De Bosscher V., Scheerder J., Ricour M., Willem A. (2020). Exercising in times of lockdown: An analysis of the impact of COVID-19 on levels and patterns of exercise among adults in Belgium. Int. J. Environ. Res. Public Health.

[26] Schwendinger F., Pocecco E. (2020). Counteracting physical inactivity during the COVID-19 pandemic: Evidence-based recommendations for home-based exercise. Int. J. Environ. Res. Public Health.

[27] Michie S., van Stralen M.M., West R. (2011). The behaviour change wheel: A new method for characterising and designing behaviour change interventions. Implement. Sci..

[28] Gibson Miller J., Hartman T.K., Levita L., Martinez A.P., Mason L., McBride O., McKay R., Murphy J., Shevlin M., Stocks T.V.A. (2020). Capability, opportunity, and motivation to enact hygienic practices in the early stages of the COVID-19 outbreak in the United Kingdom. Br. J. Health Psychol..

[29] Stockwell S., Trott M., Tully M., Shin J., Barnett Y., Butler L., McDermott D., Schuch F., Smith L. (2021). Changes in physical activity and sedentary behaviours from before to during the COVID-19 pandemic lockdown: A systematic review. BMJ Open Sport Exerc. Med..

[30] Caputo E.L., Reichert F.F. (2020). Studies of physical activity and COVID-19 during the pandemic: A scoping review. J. Phys. Act. Health.

[31] Kavaliunas A., Ocaya P., Mumper J., Lindfeldt I., Kyhlstedt M. (2020). Swedish policy analysis for Covid-19. Health Policy Technol..

[32] World Medical Association Declaration of Helsinki—Ethical Principles for Medical Research Involving Human Subjects. 2013, Adopted by the 18th WMA General Assembly, Helsinki, Finland, June 1964 (Amended by the 64th WMA General Assembly, Fortaleza, Brazil, October 2013). https://www.wma.net/policies-post/wma-declaration-of-helsinki-ethical-principles-for-medical-research-involving-human-subjects/.

[33] The Swedish Research Council Good Research Practice. https://www.vr.se/english/analysis/reports/our-reports/2017-08-31-good-research-practice.html.

[34] Public Health Agency of Sweden. https://www.folkhalsomyndigheten.se/smittskydd-beredskap/utbrott/aktuella-utbrott/covid-19/statistik-och-analyser/bekraftade-fall-i-sverige/.

[35] Craig C.L., Marshall A.L., Sjöström M., Bauman A.E., Booth M.L., Ainsworth B.E., Pratt M., Ekelund U., Yngve A., Sallis J.F. (2003). International physical activity questionnaire: 12-country reliability and validity. Med. Sci. Sports Exerc..

[36] Hurst H., Bolton J. (2004). Assessing the clinical significance of change scores recorded on subjective outcome measures. J. Manip. Physiol. Ther..

[37] Keyworth C., Epton T., Goldthorpe J., Calam R., Armitage C.J. (2020). Acceptability, reliability, and validity of a brief measure of capabilities, opportunities, and motivations (“COM-B”). Br. J. Health Psychol..

[38] Karolinska Institutet Guidelines for Data Processing and Analysis of the International Physical Activity Questionnaire (IPAQ)—Short and Long Forms. http://www.ipaq.ki.se.

[39] Field A. (2013). Discovering Statistics Using IBM SPSS Statistics.

[40] Michie S., Rubin G.J., Amlot R. Behavioural Science Must Be at the Heart of the Public Health Response to COVID-19. https://blogs.bmj.com/bmj/2020/02/28/behavioural-science-must-be-at-the-heart-of-the-public-health-response-to-covid-19/.

[41] Knittle K., Nurmi J., Crutzen R., Hankonen N., Beattie M., Dombrowski S.U. (2018). How can interventions increase motivation for physical activity? A systematic review and meta-analysis. Health Psychol. Rev..

[42] Spence J.C., Rhodes R.E., McCurdy A., Mangan A., Hopkins D., Mummery W.K. (2021). Determinants of physical activity among adults in the United Kingdom during the COVID-19 pandemic: The DUK-COVID study. Br. J. Health Psychol..

[43] Pierce M., Hope H., Ford T., Hatch S., Hotopf M., John A., Kontopantelis E., Webb R., Wessely S., McManus S. (2020). Mental health before and during the COVID-19 pandemic: A longitudinal probability sample survey of the UK population. Lancet Psychiatry..

[44] Vindegaard N., Benros M.E. (2020). COVID-19 pandemic and mental health consequences: Systematic review of the current evidence. Brain Behav. Immun..

[45] Higgins V., Sohaei D., Diamandis E.P., Prassas I. (2021). COVID-19: From an acute to chronic disease? Potential long-term health consequences. Crit. Rev. Clin. Lab. Sci..

[46] Bandura A. (1997). Self-Efficacy: The Exercise of Control.

[47] Rhodes R.E., Janssen I., Bredin S.S.D., Warburton D.E.R., Bauman A. (2017). Physical activity: Health impact, prevalence, correlates and interventions. Psychol. Health.

[48] Williams S.L., French D.P. (2011). What are the most effective intervention techniques for changing physical activity self-efficacy and physical activity behaviour—And are they the same?. Health Educ. Res..

[49] Ashford S., Edmunds J., French D.P. (2010). What is the best way to change self-efficacy to promote lifestyle and recreational physical activity? A systematic review with meta-analysis. Br. J. Health Psychol..

[50] Meyer J., McDowell C., Lansing J., Brower C., Smith L., Tully M., Herring M. (2020). Changes in physical activity and sedentary behavior in response to COVID-19 and their associations with mental health in 3052 US adults. Int. J. Environ. Res. Public Health.

[51] Chodzko-Zajko W.J., Proctor D.N., Fiatarone Singh M.A., Minson C.T., Nigg C.R., Salem G.J., Skinner J.S. (2009). Exercise and physical activity for older adults. Med. Sci. Sports Exerc..

[52] Diaz K.M., Howard V.J., Hutto B., Colabianchi N., Vena J.E., Safford M.M., Blair S.N., Hooker S.P. (2017). Patterns of sedentary behavior and mortality in U.S. middle-aged and older adults: A national cohort study. Ann. Intern. Med..

[53] Sprod J., Ferrar K., Olds T., Maher C. (2015). Changes in sedentary behaviours across the retirement transition: A systematic review. Age Ageing.

[54] Eklund C., Elfström M.L., von Heideken Wågert P., Söderlund A., Gustavsson C., Cederbom S., Thunborgh C., Lööf H. (2021). The meaning of sedentary behavior as experienced by people in the transition from working life to retirement: An empirical phenomenological study. Phys. Ther..

[55] Castañeda-Babarro A., Arbillaga-Etxarri A., Gutiérrez-Santamaría B., Coca A. (2020). Physical activity change during COVID-19 confinement. Int. J. Environ. Res. Public Health.

[56] Lanza K., Durand C.P., Alcazar M., Ehlers S., Zhang K., Kohl H.W. (2021). School parks as a community health resource: Use of joint-use parks by children before and during COVID-19 pandemic. Int. J. Environ. Res. Public Health.

[57] Tammelin T., Näyhä S., Laitinen J., Rintamäki H., Järvelin M.R. (2003). Physical activity and social status in adolescence as predictors of physical inactivity in adulthood. Prev. Med..

[58] Kerstis B., Giannotta F., Wågert P.V.H., Hellström C., Lindberg D., Stier J., Elvén M. (2021). Changes in mental health and views on communication and activities of public institutions among Swedes during the COVID-19 Pandemic—A cross-sectional repeated measures design. Healthcare.

[59] Bhopal S.S., Bagaria J., Olabi B., Bhopal R. (2021). Children and young people remain at low risk of COVID-19 mortality. Lancet Child Adolesc. Health.

[60] World Health Organization WHO Guidelines on Physical Activity and Sedentary Behaviour. https://apps.who.int/iris/bitstream/handle/10665/336656/9789240015128-eng.pdf?sequence=1&isAllowed=y.

[61] Lee P.H., Macfarlane D.J., Lam T.H., Stewart S.M. (2011). Validity of the international Physical Activity Questionnaire Short Form (IPAQ-SF): A systematic review. Int. J. Behav. Nutr. Phys. Act..

[62] Colley R.C., Butler G., Garriguet D., Prince S.A., Roberts K.C. (2018). Comparison of self-reported and accelerometer-measured physical activity in Canadian adults. Health Rep..

[63] Statistics Sweden Snabba Fakta Om Sverige. https://www.scb.se/hitta-statistik/snabba-fakta/.

