# Supplementary material for: Changes in Physical Activity and Sedentary Behavior before and during the COVID-19 Pandemic: A Swedish Population Study
Source: Int J Environ Res Public Health. 2022 Feb 23;19(5):2558. doi: 10.3390/ijerph19052558 (PMC8909725; doi:10.3390/ijerph19052558)
Supplement: Supplementary file 1 [file ijerph-19-02558-s001.zip › ijerph-1587405-supplementary.pdf]

Questionnaire about Physical Activity and Sedentary Behavior

Question 1

Think about all the **vigorous** activities that you do. **Vigorous** physical activities refer to activities that take hard physical effort and make you breathe much harder than normal like heavy lifting, digging, aerobics, running or fast bicycling. Think only about those physical activities that you do for at least 10 minutes at a time.

1a) During the **last 7 days**, on how many days did you do **vigorous** physical activities?

_____days

☐ **No vigorous physical activities -> Skip to question 1c**

1b) How much time did you usually spend doing **vigorous** physical activities on one of those days?

_____minutes per day

☐ **Don't know**

1c) During a normal week 1 year ago, on how many days did you do **vigorous** physical activities

_____days

☐ **No vigorous physical activities -> Skip to question 2**

1d) How much time did you usually spend doing **vigorous** physical activities on one of those days?

_____minutes per day

☐ **Don't know**

Question 2

Think about all the **moderate** activities that do. **Moderate** activities refer to activities that take moderate physical effort and make you breathe somewhat harder than normal like bicycling or swimming at a regular pace, carrying light loads, or gardening. Do not include walking. Think only about those physical activities that you did for at least 10 minutes at a time.

2a) During the **last 7 days**, on how many days did you do **moderate** physical activities?

_____days

☐ **No moderate physical activities -> Skip to question 2c**

2b) How much time did you usually spend doing **moderate** physical activities on one of those days?

_____hours per day
_____minutes per day

☐ **Don't know**

2c) During a normal week 1 year ago, on how many days did you do **moderate** physical activities

_____days

☐ **No moderate physical activities -> Skip to question 3**

2d) How much time did you usually spend doing **moderate** physical activities on one of those days?

_____hours per day
_____minutes per day

☐ **Don't know**

Question 3
Think about the time you spend **walking**. This includes at work and at home, walking to travel from place to place, and any other walking that you have done solely for recreation, sport, exercise, or leisure.

3a) During the **last 7 days**, on how many days did you **walk** for at least 10 minutes at a time?

_____days

☐ **No walking -> Skip to question 3c**

3b) How much time did you usually spend **walking** on one of those days?

_____hours per day
_____minutes per day

☐ **Don't know**

3c) During a normal week 1 year ago, on how many days did you **walk** for at least 10 minutes at a time?

_____days

☐ **No walking -> Skip to question 4**

3d) How much time did you usually spend **walking** on one of those days?

_____hours per day
_____minutes per day

☐ **Don't know**

Question 4
Think about the time you spend **sitting** on weekdays. Include time spent at work, at home, while studying, while transporting, and during leisure time. This may include time spent sitting at a desk, visiting friends, reading, or sitting or lying down to watch television. Normal time awake is 16 hours per day.

4a) During the **last 7 days**, how much time did you spend **sitting** on a weekday?

_____hours per day
_____minutes per day

☐ **Don't know**

4a) During a normal week 1 year ago, how much time did you spend **sitting** on a weekday?

_____hours per day
_____minutes per day

☐ **Don't know**

Question 5.

To what extent has the time you spent on **physical activity on your own, i.e., nonorganized activities** in the last 7 days, changed compared with a normal week 1 year ago? For example, exercising at home, using outdoor gyms, brisk walks, and running.

☐ Very much less time today

☐ Much less time today

☐ Slightly less time today

☐ As much time as 1 year ago or unchanged

☐ Slightly more time today

☐ Much more time today

☐ Very much more time today

☐ Don't perform this type of physical activity either now or before the COVID-19 pandemic

☐ Don't know

Question 6.

To what extent has the time you spent on **physical activity in organized activities** in the last 7 days changed compared with a normal week 1 year ago? For example, training at a fitness center or swimming hall, playing with a sports team, and rehabilitation exercises.

☐ Very much less time today

☐ Much less time today

☐ Slightly less time today

☐ As much time as 1 year ago or unchanged

☐ Slightly more time today

☐ Much more time today

☐ Very much more time today

☐ Don't perform this type of physical activity either now or before the COVID-19 pandemic

☐ Don't know

Question 7. I believe it is **important** for my well-being to perform physical activity during the COVID-19 pandemic. Physical activity refers to activities that make you breathe somewhat harder than normal.

0 1 2 3 4 5 6 7 8 9 10

Strongly disagree … Strongly agree

☐ Don't know

Question 8. Due to the COVID-19 pandemic, I feel that **my ability** to perform physical activity in the last 7 days was…

0 1 2 3 4 5 6 7 8 9 10

Much worse than 1 year ago … Unchanged … Much better than 1 year ago

☐ Don't know

Question 9. Due to the COVID-19 pandemic, I feel that my **opportunities** (e.g., access to a good environment for PA, equipment, and personal support) to perform physical activity in the last 7 days were…

0 1 2 3 4 5 6 7 8 9 10

Much worse than 1 year ago … Unchanged … Much better than 1 year ago

☐ Don't know

References:

Craig, C.L.; Marshall, A.L.; Sjöström, M.; Bauman, A.E.; Booth, M.L.; Ainsworth, B.E., . . . Oja, P. International physical activity questionnaire: 12-country reliability and validity. *Med Sci Sports Exerc.* **2003**, *35*, 1381-95.

Hurst, H.; Bolton, J. Assessing the clinical significance of change scores recorded on subjective outcome measures. *J Manipulative Physiol Ther.* **2004**, *27*, 26-35.

Keyworth, C.; Epton, T.; Goldthorpe, J.; Calam, R.; Armitage, C.J. Acceptability, reliability, and validity of a brief measure of capabilities, opportunities, and motivations ("COM-B"). *Br J Health Psychol.* **2020**, *25*, 474-501.
